# Incidence, Risk Factors, and Mortality From Hospital-Acquired Infections at a Hospital in Mauritius

**DOI:** 10.7759/cureus.19962

**Published:** 2021-11-28

**Authors:** Dooshanveer C Nuckchady

**Affiliations:** 1 Infectious Diseases, Victoria Hospital, Quatre Bornes, MUS

**Keywords:** central line-associated bloodstream infections, ventilator-associated pneumonia, surgical site infections, nosocomial, hospital-acquired infections

## Abstract

Introduction

Hospital-acquired infections can be associated with an increase in morbidity, length of stay, and cost. Data on this topic are very limited in Mauritius. This study seeks to identify (a) the most prevalent hospital-acquired infection locally, (b) the risk factors for acquiring nosocomial infections, and (c) the mortality rate linked to such infections.

Methods

This is an observational study that was conducted at a 600-bed hospital in Mauritius by going through the folders of 109 patients who were admitted in different wards. Cox regression was used to carry out the survival analysis.

Results

Over the past 25 years, the incidence of hospital-acquired infections has increased by two to three times in Mauritius to reach a value of 18 per 100 admitted patients. The most commonly identified nosocomial infection was ventilator-associated pneumonia. The presence of foreign devices increased the risk of acquiring nosocomial infections. The mortality rate from such infections was almost four times higher compared to the mortality rate from patients who did not suffer from these infections; however, after adjustment for potential confounders, this was not statistically significant. The incidence of ventilator-associated pneumonia and central line-associated bloodstream infections was high at 46 per 1,000 ventilator-days and 25 per 1,000 central line days, respectively.

Conclusion

Infection prevention and control measures should be implemented to curtail the rise of hospital-acquired infections in Mauritius. Such measures should include the use of bundles of care. In addition, periodic surveillance of nosocomial infections needs to be encouraged.

## Introduction

Hospital-acquired infections (HAIs) are associated with high morbidity and mortality; in some instances, they can lead to a prolonged length of stay at healthcare facilities, extended use of antibiotics, and an increase in readmissions [1,2]. Effective and continuous surveillance of HAIs is mandatory in order to direct appropriate infection prevention and control (IPC) practices.

The data on HAIs in low- to middle-income African countries are limited. The last published study that was carried out on HAIs in Mauritius was led by a group of Danish experts in 1993 as part of an effort by the World Health Organization to improve IPC in the country [3]. However, IPC and antimicrobial stewardship did not improve significantly, as evidenced by the elevated rate of multi-drug resistant organisms in Mauritius in 2016 and the corresponding high mortality rate of 72% [4]. The present study was an endeavor to shed light on the incidence of HAIs in Mauritius and to help inform future national IPC policies that need to be put in place to reduce deaths from HAIs.

## Materials and methods

Once weekly from June 2018 to October 2018, a trained doctor assessed the folders of patients aged ≥ 18 years who were admitted to a 600-bed hospital in Mauritius. These patients were admitted in the surgical, medical, and orthopedic wards, as well as in the surgical, medical and neurosurgical intensive care units (ICUs); each ward was surveyed on a different day. After their discharge, the medical records were reevaluated to gather data regarding discharge outcomes.

In order to reduce selection bias, equal representation was provided to each of the wards, i.e., for the purpose of calculating the incidence of HAIs, wards could not be represented more than once. Moreover, to reduce differences between groups, for the calculations involving risk factors and outcomes, the control group consisted of patients who were diagnosed with community-acquired infections as per their treating doctor.

The objectives of the study were three-fold: (1) to identify the incidence of HAIs, (2) to determine the risk factors that are associated with HAIs, and (3) to describe the mortality rate associated with HAIs. A p-value of less than 0.05 was considered significant.

Data analysis was conducted using Excel version 2104 (Build 13929.20386 Click-to-Run; Microsoft, Redmond, WA), R version 3.3.1 (R Foundation for Statistical Computing, Vienna, Austria), and IBM SPSS Statistics version 20 (IBM Corp., Armonk, NY). Categorical variables were compared using the odds ratio, and Student’s t-test was used to analyze continuous variables. The Kaplan-Meier method and Cox regression analysis were utilized to assess mortality rates. Adjustment for confounders was also performed; confounders were chosen only when they were biologically plausible and when more than 10 events were present for that confounder in the data set.

The case definitions of hospital-acquired pneumonia (HAP), surgical site infections (SSI), ventilator-associated pneumonia (VAP), central line-associated bloodstream infections (CLABSI), and catheter-associated urinary tract infections (CAUTI) that were used were based on the USA’s National Healthcare Safety Network’s definitions [5-9]. However, these definitions had to be modified to suit the local context; Table 1 elaborates on these criteria (see the supplementary Appendix for more details).

**Table 1:** Definitions of site-specific hospital-acquired infections. CAUTI, catheter-associated urinary tract infections; CLABSI, central line-associated bloodstream infections; CXR, chest X-ray; HAI, hospital-acquired infections; HAP, hospital-acquired pneumonia; PCR, polymerase chain reaction; SSI, surgical site infection; US, ultrasound; VAP, ventilator-associated pneumonia; WBC, white blood cells

HAI	Timeline	Clinical	Laboratory	Radiological	Microbiological
VAP	>48 hours after intubation or <48 hours after extubation	Fever > 38.0°C or < 36°C, new antimicrobial started, hypoxia, septic shock without any other cause	WBC ≤ 4,000/mm^3^ or WBC ≥ 12,000/mm^3^	CXR or CT chest	Endotracheal aspirate culture, pleural fluid culture, urine antigen test, or PCR on respiratory samples
CLABSI	Line is present > 48 hours or line is removed < 24 hours ago	If a commensal is grown: fever > 38°C, chills, hypotension			Blood culture
SSI	D0 to D30 after most surgeries without foreign bodies being present; D0 to D90 if foreign bodies are inserted	Inflammation at surgical site, pus, dehisced wound, localized pain or tenderness, localized swelling, erythema around the site, heat at the site or fever > 38°C		US/ CT/MRI showing infection at surgical site	Pus culture
CAUTI	Foley is present > 48 hours or Foley is removed < 24 hours ago	Fever > 38°C, suprapubic tenderness or costovertebral angle tenderness			Urine culture
HAP	> 48 hours after admission	Fever > 38.0°C, confused if ≥ 70 years old, cough, dyspnea, bronchial breathing, or hypoxia	WBC ≤ 4,000/mm^3^ or WBC ≥ 12,000/mm^3^	CXR or CT chest	Sputum culture, urine antigen test, or PCR on respiratory samples

The analytical profile index and matrix-assisted laser desorption ionization time-of-flight mass spectrometry were used to identify bacteria grown in cultures. The Kirby-Bauer technique and the E-test were applied to assess for antibiotic resistance. The Clinical & Laboratory Standards Institute’s MIC (minimal inhibitory concentration) threshold was adopted in this study.

Approval was obtained from the Ethics Committee of the Ministry of Health and Wellness of Mauritius to carry out this research.

## Results

**Figure 1:**
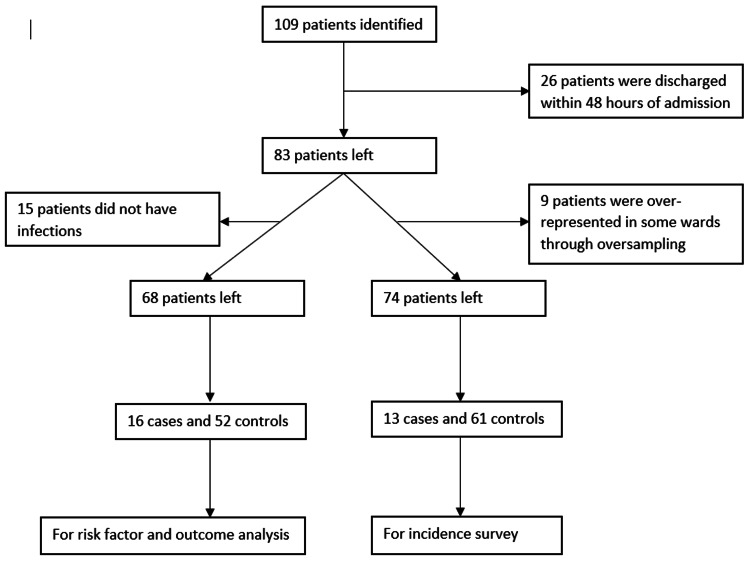
Flow diagram illustrating how patients were selected in the study.

The folders of 109 patients were assessed; 26 patients were excluded because they did not meet the study’s inclusion criteria; in particular, they were discharged too early to have been able to develop an HAI (see Figure 1 for details).

Table 2 lists the characteristics of the patients included in the study. The mean age of the participants was 57 years (58 years among controls and 53 years among cases, with p = 0.41), the mean length of stay in the hospital was 29 days (21 days among controls and 53 days among cases, p = 2.0E-7), and their mean sequential organ failure assessment (SOFA) score was 2.1 (1.5 among controls and 3.9 among cases, p = 8.2E-4).

**Table 2:** Main characteristics of the controls and cases. CI, confidence interval; HAI, hospital-acquired infections; HCF, healthcare facility; IVDU, intravenous drug user; OR, odds ratio; SOFA, sequential organ failure assessment

Variable	Controls (n = 52)	Cases (n = 16)	OR (95% CI)	p-Value
Males	32 (62%)	7 (44%)	0.49 (0.16–1.5)	0.21
Age ≥ 60 years	27 (52%)	4 (25%)	0.31 (0.09–1.1)	0.05
SOFA > 2	10 (19%)	12 (75%)	13 (3.3–47)	1.8E-4
Central venous line present	8 (15%)	9 (56%)	7.1 (2.0–24)	0.002
Hemodialysis line present on admission	2 (3.8%)	1 (6.3%)	1.7 (0.14–20)	0.69
Foley catheter present	20 (38%)	11 (69%)	3.5 (1.1–12)	0.04
On ventilator	4 (7.7%)	6 (38%)	7.2 (1.7–30)	0.007
Surgery done during admission	18 (35%)	10 (63%)	3.1 (1.0 - 10)	0.05
Diabetes mellitus	10 (19%)	5 (31%)	1.9 (0.54–6.7)	0.32
Cerebrovascular accident	7 (13%)	1 (6.3%)	0.43 (0.05–3.8)	0.45
Dialyzed at baseline	1 (1.9%)	1 (6.3%)	3.4 (0.20–58)	0.40
Asthma	2 (3.8%)	0 (0%)	-	1.0
Cirrhosis	1 (1.9%)	0 (0%)	-	1.0
Congestive heart failure	1 (1.9%)	1 (6.3%)	3.4 (0.20–58)	0.40
Ischemic heart disease	4 (7.7%)	2 (13%)	1.7 (0.28––10)	0.56
Peripheral vascular disease	0 (0%)	0 (0%)	-	1.0
Cancer	3 (5.8%)	1 (6.3%)	1.1 (0.11 - 11)	0.94
Admitted in an HCF in the last 3 months	16 (31%)	5 (31%)	1.0 (0.31–3.4)	0.97
Decubitus ulcers	0 (0%)	2 (13%)	-	1.0
On steroids	2 (3.8%)	4 (25%)	8.3 (1.4–51)	0.02
On chemotherapy in the last 6 months	2 (3.8%)	1 (6.3%)	1.7 (0.14 - 20)	0.69
HIV status	4 (7.7%)	1 (6.3%)	0.80 (0.08–7.7)	0.85
Malnourished	1 (1.9%)	0 (0%)	-	1.0
IVDU	5 (9.6%)	1 (6.3%)	0.63 (0.07–5.8)	0.68
On appropriate empiric antibiotics initially	15 (29%)	1 (6.3%)	0.16 (0.02–1.4)	0.09
Inotropes started	1 (1.9%)	3 (19%)	12 (1.1–120)	0.04
Started dialysis after onset of HAI	1 (1.9%)	1 (6.3%)	3.4 (0.20–58)	0.40
Mean length of stay (days)	21	53	-	2.0E-7
Deaths	7 (13%)	8 (50%)	6.4 (1.8 - 23)	0.004

For the incidence survey, 13 patients were recorded as having an infection that was not incubating upon admission; this gave an incidence risk of HAI of 18 per 100 patients. The incidence risk of HAI in the ICU was 44 per 100 patients while that in the general wards was 9 per 100 patients.

Using the pre-determined case definitions, the incidence of HAP (including VAP), SSI, VAP, CLABSI, and CAUTI were determined to be 9.5, 17, 63, 36, and 13 per 100 at-risk patients, respectively. The device-associated infection rates were as follows: 46 VAP per 1,000 ventilator-days, 25 CLABSI per 1,000 central line days, and 8 CAUTI per 1,000 Foley catheter days.

For the risk factor analysis, 68 patients had an infection out of 109. The risk factors that were associated with HAIs in this study were SOFA score, length of stay in the hospital, presence of a central venous catheter, presence of a Foley catheter, being on a ventilator, being on steroids, and being on pressors.

**Figure 2:**
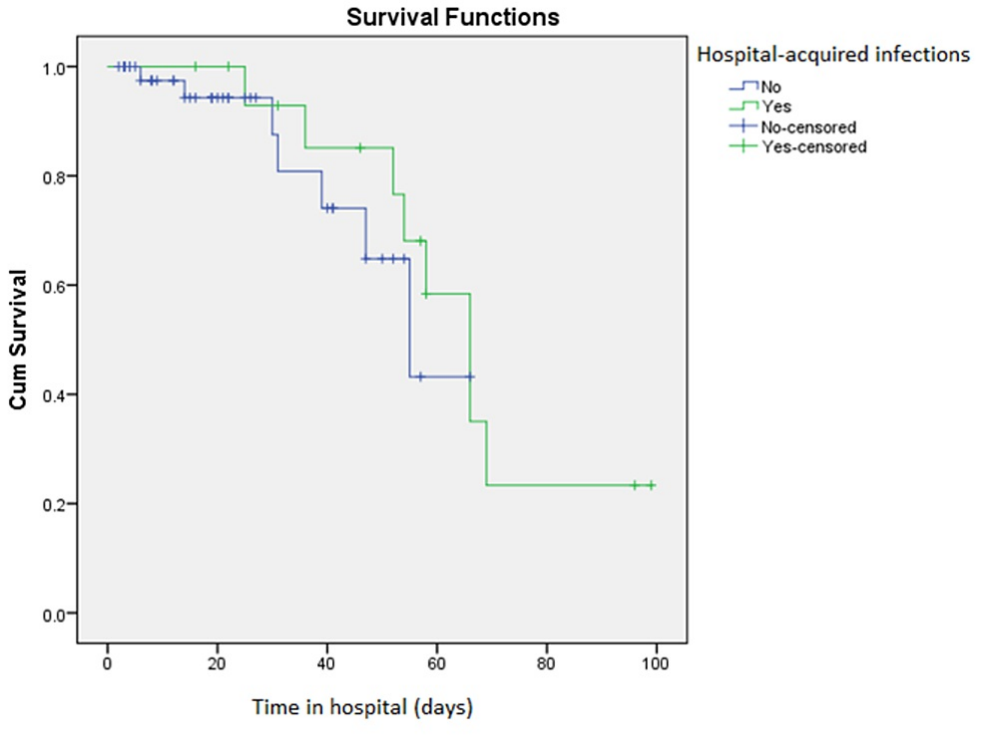
The Kaplan-Meier curve demonstrating the survival of patients with and without hospital-acquired infections.

During the course of this study, 15 patients died. The mortality rate among patients who did not develop an HAI was 13%, while the mortality rate among those with an HAI was 50% (p = 0.004). However, after adjustment for age and ventilation status, HAI was no longer associated with death according to the Cox regression analysis (p = 0.26). Figure 2 shows the corresponding Kaplan-Meier curves. Of note, 100% of patients who were intubated for more than 48 hours died. Moreover, the mortality rate of HAI cases in the ICU was 73%, while that in the general wards was 0%.

The mortality rates of patients who developed HAP (excluding VAP), SSI, VAP, CLABSI, or CAUTI compared to those who did not develop these HAIs were 100% vs 16%, 43% vs 24%, 100% vs 80%, 80% vs 69%, and 67% vs 40%, respectively.

Among the 124 organisms that were cultured in the HAI group (excluding coagulase-negative staphylococcus and diphtheroids), 26% were *Acinetobacter baumannii* (of which 100% were carbapenem-resistant), 22% were *Klebsiella pneumoniae* (of which 93% were carbapenem-resistant), 11% were *Escherichia coli* (of which 43% were extended-spectrum beta-lactamase producers), 10% were *Enterococcus* sp. (of which 0% were vancomycin-resistant), and 10% were *Pseudomonas aeruginosa* (of which 75% were carbapenem-resistant). The odds of acquiring a multi-drug resistant organism when developing an HAI was 1.9 (95% CI: 0.68-5.5; p = 0.22).

**Figure 3:**
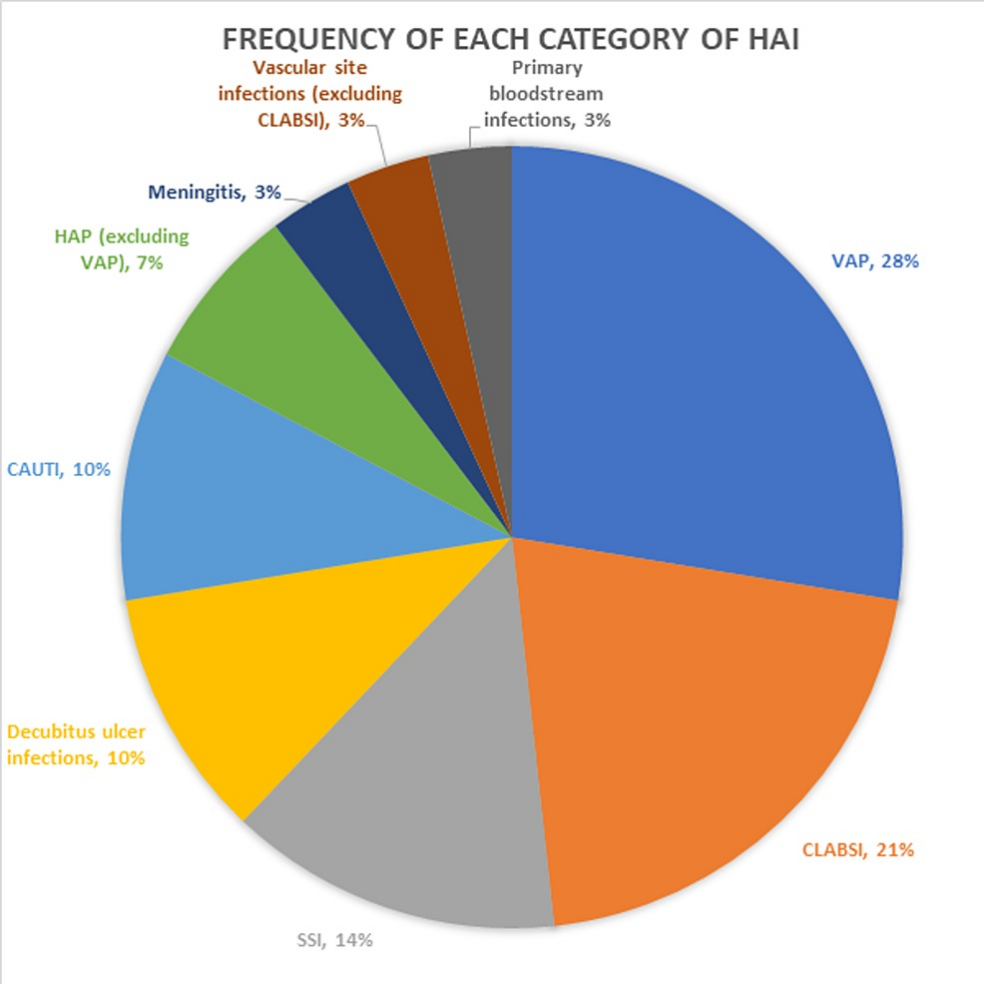
Pie chart classifying the frequency of each HAI that was identified in the study. VAP, CLABSI, and SSI were the most common types of HAIs, while primary bloodstream infections and vascular site infections were the rarest. CLABSI, central line-associated bloodstream infections; HAI, hospital-acquired infections; SSI, surgical site infection; VAP, ventilator-associated pneumonia

**Figure 4:**
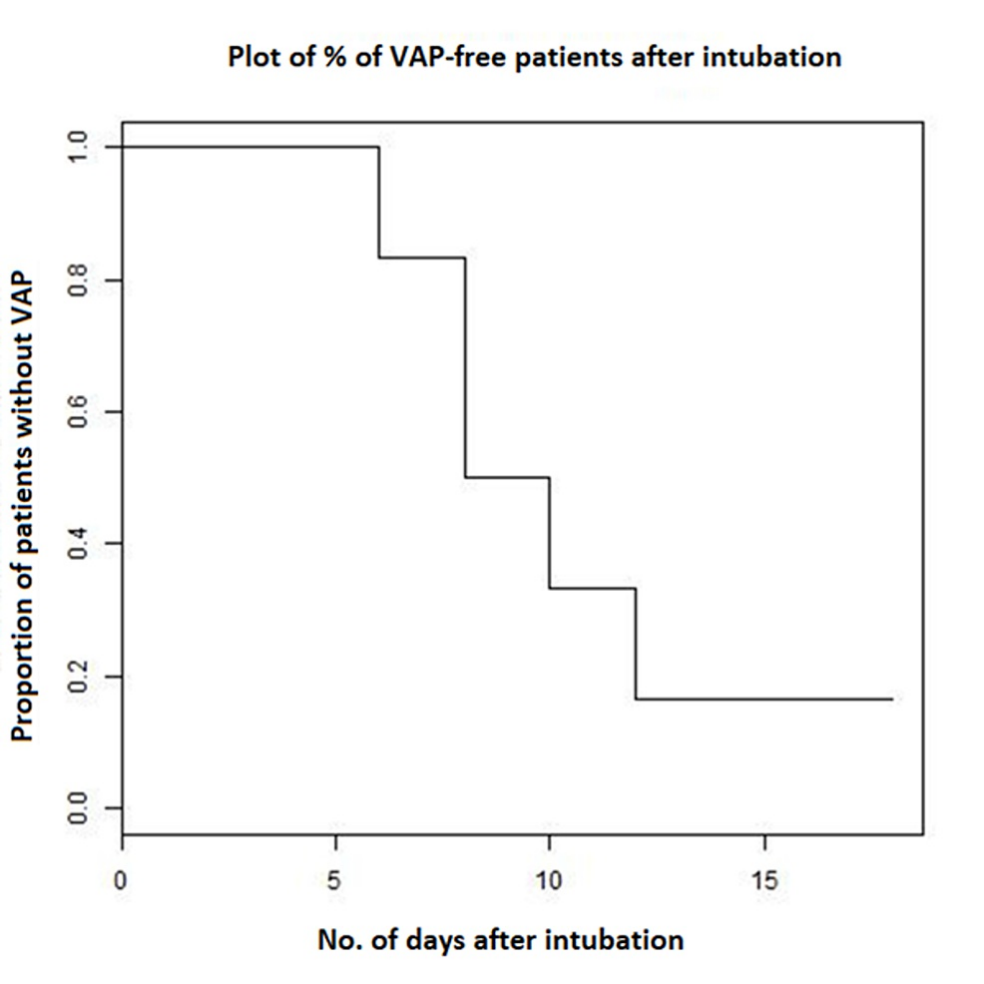
The Kaplan-Meier curve illustrating the risk of developing VAP after being intubated. By 18 days, only 17% of intubated patients did not develop a VAP yet. VAP, ventilator-associated pneumonia

As a proportion of the total number of HAIs identified, the most common type of HAI was VAP (28%), followed by CLABSI (21%) (see Figure 3 for details). Also, 83% of patients who were intubated for more than five days developed a VAP (see Figure 4 for the corresponding Kaplan-Meier curve).

## Discussion

Given the low level of hand hygiene compliance rate (ranging from 1% to 12%) in some of the hospitals in Mauritius [10], it is not surprising that the incidence of HAIs and SSI increased from 4.9 and 8.2 per 100 patients in 1993 to 18 and 17 per 100 patients, respectively, in 2018 [3]. However, one has to be careful when comparing these studies since the case definitions may have been different.

According to the World Bank, as of July 1, 2020, Mauritius became a high-income country [11]; hence, it is reasonable for the country to compare itself with other neighboring high-income nations. Compared to Singapore, the rate of HAIs in Mauritius is 34% higher [12]. In addition, in contrast to South Africa, the incidence risk of HAIs is almost twice higher in our study [13].

Likewise, in the United States, the incidence of VAP was six times lower than that in Mauritius [14]; this finding suggests that IPC measures in ventilated patients should be reinforced. In fact, an audit carried out at the end of 2020 showed that (a) 63% of intubated patients had a Richmond Agitation and Sedation Scale of less than -1, thus indicating oversedation, (b) 30% of ventilated patients did not have their heads of bed elevated at 30°-45°, (c) 0% of patients had routine mouth care using chlorhexidine, and (d) 78% of patients were prescribed proton pump inhibitors as ulcer prophylaxis.

Similarly, the rate of CLABSI was 25 times higher in this study compared to data from Australia [15]; the central line bundle of care should be urgently implemented in Mauritius in order to save patient lives. The same audit in 2020 demonstrated that (a) all central lines were inserted without the help of ultrasound devices, (b) transparent dressings were not in use, (c) no nursing checklist was utilized to ensure that catheter sites were checked daily for signs of infection, and (d) patients were not bathed (especially around the catheter site) with chlorhexidine daily.

In Saudi Arabia, the incidence of SSI was only 6.8 per 100 patients, i.e., 2.5 times lower than the rate found in our cohort of patients [16]. The corresponding rate in Iran was even lower at 2.4 per 100 cases [17]. Of note, in our study, the patients were followed for only a mean of 13 days post-surgery; through the use of logistic regression, had the patients been followed for 30 days, the rate of SSI would have been much higher at 39 per 100 cases. This is not surprising since as per the audit of 2020, (a) 67% of patients received their antibiotic prophylaxis more than 2 hours before surgery, (b) 100% of diabetic patients had abnormally high glucose levels peri-operatively, (c) none of the patients had their temperatures recorded intra-operatively, and (d) 100% of patients had their hairs shaved instead of clipped. Furthermore, 85% of patients had their antibiotics continued for more than 24 hours after surgery.

The risk factors associated with HAI included the presence of foreign bodies such as central venous lines and bladder catheters. This has been noted in several studies previously and underlines the importance of removing foreign devices as soon as medically feasible in order to reduce the risk of contracting an HAI.

The exceedingly high resistance rates of bacteria among patients with HAIs is not surprising given that in 2016, amidst patients admitted to the ICU, 86% of *Acinetobacter* spp., 30% of *Enterobacteriaceae*, and 80% of *Pseudomonas* spp. were carbapenem-resistant [4]. The corresponding values in this study are suggestive of a general escalation in antibiotic resistance over a period of two years from 2016 to 2018.

The increase in length of stay by 2.5 times is concerning since this can lead to a rise in hospital-related costs. This has been confirmed in multiple other studies previously [2].

The strikingly high mortality rate among ventilated patients has also already been described in a recent study in Mauritius [4]. Even after adjustment for the SOFA score, the mortality rate was two to three times higher when compared to that in developed countries. One of the main causes of death could be VAP; in fact, most countries have an incidence of VAP of 5 to 40 per 100 intubated patients [18], which suggests that the incidence of 63 per 100 cases found in this study is one of the highest in the world.

HAI was associated with a 3.8 times increased risk of dying. However, due to the small size of the study and its limited power, statistical significance could not be reached. Other limitations of the study include the fact that it is a single-center study, patients were not followed for a total of 30 days post-operatively, and the rate of SSI was not categorized by the type of surgery performed.

Nonetheless, this study succeeds in showing that African countries can carry out incidence surveys for HAIs through the use of locally adapted definitions for nosocomial infections. It is hoped that surrounding countries will follow suit and more data on HAIs in the African continent will become available in the near future.

## Conclusions

This study highlights the rising incidence of HAIs in Mauritius and sounds the alarm about the high mortality associated with HAIs. The author recommends that corrective measures should be implemented expeditiously and that studies on the surveillance of HAIs should be carried out more regularly on a larger sample size. Once the Ministry of Health and Wellness took note of the findings in this study, a decision was taken to write the first national guidelines on IPC for Mauritius. Hopefully, new protocols will guide the implementation of evidence-based bundles of care that will eventually reduce the prevalence of HAIs in the country.

## Appendices

**Table 3:** Definitions of hospital-acquired infections in adults. CAUTI, catheter-associated urinary tract infections; CLABSI, central line-associated bloodstream infections; CXR, chest X-ray; DOE, date of event; DOD, date of diagnosis; HAI, hospital-acquired infections; HAP, hospital-acquired pneumonia; PCR, polymerase chain reaction; PEEP, positive end-expiratory pressure; SSI, surgical site infection; VAP, ventilator-associated pneumonia; WBC, white blood cell

Terms	Definitions
Contaminants, colonizers, or commensals	Coagulase-negative staphylococcus, diphtheroids, *Candida* sp. and *Bacillus* sp.
	For infections that require positive blood cultures for their diagnoses, the presence of *Candida* sp. in the blood culture is not considered a contaminant.
DOE	The date when the first element used to meet the infection criteria occurs.
Infection window period	All the criteria for the infection must be met within the 7-day Infection Window Period (3 days before the date of the first diagnostic test (DOD), on the DOD or 3 days after the DOD).
	A diagnostic test is a laboratory, radiological, or microbiological test that is part of the list of criteria used for the diagnosis of the specific hospital-acquired infection.
	The infection window period cannot start at a time when the patient was not at risk for the infection (e.g., ≤ 48 hours after intubation).
Reactivation	Reactivation of latent disease such as tuberculosis is not considered a hospital-acquired infection.
Repeat infection timeframe	A repeat event within 14 days is considered as persistence or relapse of the same infection and is not counted twice.
	However, an exception is made if both of the following are present: the patient’s diagnostic test is positive for a new organism which is neither a contaminant nor a colonizer AND there is at least one new infection criterion that is met which was not present in the last 48 hours (e.g., for a diagnosis of pneumonia, the patient develops new-onset hypoxia which was absent previously). Under such circumstances, a new event is considered to have occurred.
Incidence	Formulae
Incidence risk of HAI	No. of patients with at least one new diagnosis of HAI / No. of patients who could get an HAI * 100
	No. of patients who could get an HAI = no. of patients admitted for > 48 hours to the hospital and no. of patients who had surgery in the first 48 hours (if any).
	For all calculations of incidence risks, readmissions of the same patients are ignored.
Incidence risk of HAP	No. of patients with at least one new HAP diagnosis / No. of patients admitted to the hospital for more than 48 hours * 100
Incidence risk of SSI	No. of patients with at least one new SSI diagnosis / No. of patients who underwent surgery * 100
Incidence risk of VAP	No. of patients with at least one new VAP diagnosis / No. of patients intubated for more than 48 hours * 100
Incidence rate by device-days of VAP	No. of VAP events / No. of ventilator days * 1,000
	No. of ventilator days includes the first 48 hours of ventilation.
Incidence risk of CLABSI	No. of patients with at least one new CLABSI / No. of patients with one or more central lines inserted for more than 48 hours * 100
Incidence rate by device-days of CLABSI	No. of CLABSI events / No. of central line days * 1,000
	No. of central line days includes the first 48 hours after insertion of the line.
Incidence risk of CAUTI	No. of patients with at least one new episode of CAUTI in that admission / No. of patients with a urinary catheter inserted for more than 48 hours * 100
Incidence rate by device-days of CAUTI	No. of CAUTI events / No. of urinary catheter days * 1,000
	No. of urinary catheter days includes the first 48 hours after insertion of the Foley.
HAI	Definition
HAP	DOE is > 48 hours after admission. PLUS
	One chest X-ray or chest CT is consistent with pneumonia. If a chest X-ray or chest CT is not done, all of the following should be present: a new antibiotic is started for ≥ 4 days AND no other source of infection is found AND a third criterion from the list marked with a asterisk (*) below is observed. PLUS
	At least one of the following: fever (> 38.0°C / 100.4°F), leukopenia (≤ 4,000 WBC/mm^3^) or leukocytosis (≥ 12,000 WBC/mm^3^), altered mental status with no other cause in ≥ 70 years old. PLUS
	At least two of the following: new onset of purulent sputum, or change in character of sputum, or increased respiratory secretions, or increased suctioning requirements; new onset or worsening cough, dyspnea, or tachypnea; rales or bronchial breath sounds; worsening gas exchange (e.g., oxygen desaturation), O_2_ requirement, or increased ventilation demand - defined as PaO_2_/FiO_2_ < 240, SpO_2_ < 94% on room air or need for supplemental oxygen; positive sputum culture (or other respiratory sample) or pleural fluid culture with an organism other than a contaminant or colonizer; positive urine antigen test for Legionella sp. or Streptococcus pneumoniae; positive PCR test on sputum (or other respiratory sample), throat swab, or nasopharyngeal swab for an organism that is not a contaminant nor a colonizer.*
	Note that patients who meet criteria for ventilator-associated pneumonia (see below) are considered to have HAP too.
SSI	Symptoms start from day 0 to day 30 after most surgeries or within 90 days after surgeries where foreign bodies are inserted. PLUS
	Involvement of skin, subcutaneous tissues, fascia, muscle, or viscera close to the surgical site. PLUS
	At least one of the following is present: purulent discharge from the site, positive culture for a non-commensal from a swab, a drain or an aspirated abscess, (incision was opened by a doctor or the wound dehisced with at least one of the following: localized pain or tenderness, localized swelling, erythema around the site, heat at the site, fever > 38°C), a diagnosis of surgical site infection as recorded by a doctor in the patient chart, a radiological imaging study that demonstrates the presence of an abscess near the surgical site.
	Note that a stitch abscess with minimal discharge and erythema is not considered an SSI and that the presence of infection at the time of surgery does not preclude the diagnosis of SSI. Patients who underwent multiple surgeries during the same admission are counted a single time. For the same surgery, an SSI cannot recur.
VAP	DOE is > 48 hours after intubation or < 48 hours after extubation. PLUS
	One chest x-ray or chest CT is consistent with pneumonia. PLUS
	Both of the following are present: (temperature > 38°C or < 36°C, OR WBC count ≥ 12,000 cells/mm^3^ or ≤ 4,000 cells/mm^3^) AND a new antimicrobial agent is started for ≥ 4 days (the same or different combinations of antibiotics may be used during these 4 days). PLUS
	At least one of the following is present: new onset or worsening hypoxia as defined by a drop of oxygen saturation (by ≥ 3% in SpO_2_ for the same FiO_2_) or a rise in FiO_2_ requirement (by ≥ 20%) or an increase in PEEP (by ≥ 3 cmH_2_O); new-onset septic shock with no other cause is recorded by the treating physician; an endotracheal aspirate (or other respiratory sample) or pleural fluid culture that is positive for a non-commensal; positive urine antigen test for Legionella sp. or Streptococcus pneumoniae; positive PCR test on endotracheal secretion, throat swab, or nasopharyngeal swab for an organism that is a non-commensal.
	Note that since most of the diagnoses of VAP in the study depended on culture results and it can take 5 to 7 days for doctors to receive culture results at that institution, the infection window period for VAP was increased to 15 days, i.e., 7 days before and 7 days after the DOE. Also, FiO_2_ and PEEP are not the main criteria for the diagnosis of VAP in the study since these are rarely recorded in a consistent manner in the charts.
CLABSI	Central line is present for > 48 hours or was removed < 24 hours ago. PLUS
	The identified organism is not related to an infection at another site. PLUS
	At least one of the following: a positive blood culture for a non-commensal or ≥ 2 positive blood cultures for a commensal in a patient that has at least fever > 38°C, chills, or hypotension.
	Note that a non-bacteremia catheter-related infection or vascular site infection is said to have occurred if a tip culture or pus culture at the site of insertion of the device is positive for a non-commensal and if at least one of the following is present: purulent discharge at the catheter site or the resolution of fever > 38°C, chills, or hypotension 48 hours after removal of the catheter.
CAUTI	Urinary catheter is present for > 48 hours or was removed < 24 hours ago. PLUS
	At least one of the following: fever > 38°C, suprapubic tenderness without any other cause, costovertebral angle pain, or tenderness without any other cause. PLUS
	A urine culture with at most two organisms that are not commensals.
